# 骨与软组织肉瘤肺转移的单中心大样本外科治疗结果及预后分析

**DOI:** 10.3779/j.issn.1009-3419.2016.05.10

**Published:** 2016-05-20

**Authors:** 晓征 康, 万璞 闫, 永波 杨, 亮 戴, 震 梁, 真 黄, 晓辉 牛, 克能 陈

**Affiliations:** 1 100142 北京，北京大学肿瘤医院暨恶性肿瘤发病机制及转化研究教育部重点实验室，胸外一科 Key Laboratory of Carcinogenesis and Translational Research (Ministry of Education), the First Department of Thoracic Surgery, Peking University Cancer Hospital and Institute, Peking University School of Oncology, Beijing 100142, China; 2 100035 北京，北京积水潭医院骨肿瘤科 Department of Orthopedic Oncology, Beijing Jishuitan Hospital, Peking University, Beijing 100035, China

**Keywords:** 骨肉瘤, 软组织肉瘤, 转移, 复发, 预后, Osteosarcoma, Soft Tissue Sarcoma, Metastasis, Recurrence, Prognosis

## Abstract

**背景与目的:**

肺是骨与软组织肉瘤最常见的远隔转移脏器，肺转移严重影响患者长期生存。肺转移瘤切除术有助于改善预后，然而对其临床地位、适应证及预后影响因素的认识目前仍存在争议。由于发病率较低难以开展随机对照研究，同时国际单中心大宗病例回顾研究也极为罕见，国内尚无类似报道。本研究旨在回顾本组单中心大样本肺转移性骨与软组织肉瘤的外科治疗结果，并且对预后影响因素进行分析。

**方法:**

2007年1月-2015年12月期间，经病理确诊为骨与软组织肉瘤，已在多学科综合治疗框架下完成原发病灶根治性切除，并且至少经过1次肺转移瘤切除术的所有患者均纳入分析。收集相关临床变量，运用*Cox*风险比例回归法进行单因素及多因素分析寻找与预后影响因素。

**结果:**

144例骨与软组织肉瘤患者符合纳入标准，总共行155次肺转移瘤切除术。多因素分析结果提示非R0切除、无病间期 < 1年、肺转移灶数目≥3枚、肺转移灶的长径总和≥45 mm均是预后的独立危险因素。

**结论:**

积极行肺转移瘤外科治疗有助于改善转移性骨与软组织肉瘤患者的长期预后。R0切除，无病间期时间较长，转移瘤数目较少及长径总和较小是本组患者良好的预后因素。

## Abstract (en)

**Background and objective:**

The bone and soft tissue sarcoma can metastasize to distant sites, most commonly the lungs. Some cases can be cured by radical metastasectomy, but its role, indication and prognostic factors remains controversial. The rarity of the disease combined with the diverse number of subtypes can make bone and soft tissue sarcomas very difficult to study. There are few randomized control studies or international high volume results, and such reports in China are seldom seen. The aim of this study is to investigate surgical treatment outcomes and prognostic factors of pulmonary metastatic bone and soft tissue sarcoma patients.

**Methods:**

From January 2007 to December 2015, patients with bone and soft tissue sarcoma who underwent multimodality therapy including definitive surgery for the primary lesion and at least one pulmonary metastasectomy were enrolled in the retrospective study. All the relevant clinical variables were collected, and then statistically analyzed and interpreted with the aid of univariate and multivariate *Cox* proportional hazard regression method.

**Results:**

Totally 155 pulmonary metastasectomies in 144 patients were analyzed. Incomplete R0 resection, a less than 1-year interval from a previous surgery, more than three detected nodules; and the summed maximum diameter of more than 45 mm for pulmonary metastases were independent prognostic indicators by multivariate analysis.

**Conclusion:**

We suggest that metastatic bone and soft tissue sarcoma patients can benefit most from aggressive surgical intervention of pulmonary metastasectomy. Its prognostic factors include R0 resection, a longer interval from a previous surgery, smaller total number and total size of pulmonary metastases.

骨与软组织肉瘤属于罕见疾病^[1-4]^，骨肉瘤仅占成人所有癌种的0.2%，占儿童癌种的5%，软组织肉瘤所占比例也不足1%，其发病率具有两个高峰期，包括青少年及70岁以上老年^[5-7]^。骨肉瘤是最常见的原发性骨与软组织肉瘤（约占35%），其次为软骨肉瘤（25%），Ewing肉瘤（16%）及软骨瘤（8%）；平滑肌肉瘤是最常见的软组织肉瘤（23.9%）。如上肿瘤不但罕见，而且诊治效果并不令人满意，骨与软组织肉瘤一旦发生转移疗效极差，危害极大^[8-10]^，而肺转移是骨与软组织肉瘤的主要死因，危害又首当其冲，所以受到普遍关注。约10%-15%骨肉瘤^[11, 12]^及20%软组织肉瘤^[13]^患者确诊时已发生远处转移，以后疗程中或随访中发生转移率约30%-40%^[14]^，其中肺转移约占90%。骨与软组织肉瘤肺转移的治疗虽然包括多学科方法^[9, 10, 15-19]^，但手术切除仍然是主要手段，备受推崇^[17, 20-23]^。遗憾的是，对于肺转移瘤切除术的地位、适应证及预后影响因素的认识目前仍存在很大差异，究其原因在于发病率低又难以进行大规模多中心随机对照临床研究^[24]^。迄今为止全球仅有意大利Rizzoli骨科中心^[25]^、美国M.D. Anderson癌症中心^[26]^、美国德克萨斯州医学中心^[27]^及美国Roswell Park癌症中心^[28]^四项单中心大宗病例回顾报道，得出的结论也需要更多的验证。

北京大学肿瘤医院胸部肿瘤中心是中国同行中的优质团队之一，团队掌握着最先进的胸外科技术，并有多年形成胸部肿瘤多学科协作机制。而北京积水潭医院骨肿瘤科则是全球最大的骨与软组织肉瘤专科之一，集中有世界上数量最多的骨与软组织肉瘤病例。自从2005年以这两个团队为主组成骨与软组织肉瘤肺转移多学科协作团队，迄今已治疗超过250例骨与软组织肉瘤肺转移患者。本组不仅是国内及亚洲最大的，即便在全球也是排名第三位的大宗骨与软组织肉瘤肺转移报道。本研究旨在回顾本组肺转移性肉瘤的外科治疗结果，并且对预后影响因素进行分析。

## 1 资料与方法

### 1.1 研究对象

回顾北京大学肿瘤医院胸外一科肺转移瘤前瞻性数据库资料，2007年1月1日-2015年12月31日所有经手术治疗的骨与软组织肉瘤肺转移患者均纳入分析。

### 1.2 纳入标准

① 经病理学确诊的骨与软组织肉瘤，并且原发灶已行根治性切除术，无局部复发征象；②曾行至少1次肺转移瘤切除术，病理确诊为转移性骨与软组织肉瘤；③术前影像学评估除外肺外转移，经多学科讨论确定存在肺内病灶根治性切除（R0）可能性，预计心肺代偿功能可耐受手术者。

### 1.3 排除标准

① 存在肺外转移灶，或双肺广泛播散转移，无法行根治性切除，仅行活检手术者；②心肺代偿功能无法耐受根治性切除，仅行活检手术者。

### 1.4 临床分析变量

包括患者年龄、性别、原发灶病理类型、肺转移瘤分布（单侧或双侧肺）、肺内病灶发现时机（原发灶初诊时，初步治疗期间或随访过程中）、手术切除方式（胸腔镜辅助方式或开放式）、切除状态（R0、R1或R2）、无病间期、肺切除术次数、肺内转移瘤总数及长径总和。

### 1.5 研究定义

无病间期的定义为原发灶根治性切除术日期至首次发现肺内病灶日期，初诊时同时性肺转移病例的无病间期为0。总体生存期（overall survival, OS）定义为肺切除术日期至全病因死亡或失访日期。无疾病生存期（disease free survival, DFS）定义为肺切除术日期至确诊局部复发或远处转移日期，或失访日期。R0切除状态的定义为术前影像学检出的所有肺转移瘤（单侧或双侧肺）经外科治疗1个月内被全部清除。计划分期手术治疗双肺转移瘤视为一次肺转移瘤治疗。肺转移灶数目以病理诊断证实来源于骨与软组织肉瘤的结果为准。“预后良好”与“预后不良”两个亚组根据经过多因素分析确定的所有独立预后影响因素定义，“预后良好”组定义为满足所有独立的预后保护性因素，对照组则定为“预后不良”组。

### 1.6 随访方式

患者遵循严格胸部计算机断层扫描（computed tomography, CT）影像学随访，即初诊时、术前及术后2年内每3个月复查1次，术后第3、4年每6个月复查1次，术后第5年及以后每12个月复查1次，直至再次复发或全病因死亡。本组随访截止日期为2016年4月30日。

### 1.7 统计学方法

生存分析采用*Kaplan*-*Meier*乘积法及*Log*-*rank*检验。*Cox*比例风险回归模型计算各临床变量与预后的关系。*P* < 0.05认为差异具有统计学意义，所有统计学方法均选择双侧检验。运用软件JMP^®^ Pro（v12.0.1, SAS Institute, Cary, NC, USA）完成统计学分析。

## 2 结果

2007年1月1日-2015年12月31日期间，北京大学肿瘤医院胸外一科共收治197例肺转移性骨与软组织肉瘤患者，其中原发灶在北京积水潭医院骨肿瘤科诊治者184例（95.9%），最终符合纳入标准者144例，一般情况及变量见表 1。其中肺转移瘤切除术前行全身化疗者39例（27.1%），观察期超过3个月者11例（7.6%），术后进一步行全身化疗者128例（88.9%）。所有患者行肺转移瘤切除术总共155次，其中133例（92.4%）仅行1次肺转移瘤切除术，11例（7.6%）行2次肺转移瘤切除术。首次切除的肺转移瘤长径总和的中位数为15 mm（5 mm-114 mm），再次切除的肺转移瘤长径总和的中位数为16 mm（3 mm-93 mm）。

**1:** 骨与软组织肉瘤肺转移患者的一般资料及临床变量 Demographics and clinical variables of patients diagnosed with pulmonary metastatic bone and soft tissue sarcoma

Items	*n*
Age (median)(yr)	9-67 (23)
Gender	
Male	108
Female	36
Histology classification	
Osteosarcoma	107
Soft tissue sarcoma	37
Affected laterality	
Left sided	50
Right sided	47
Bilateral	47
Timing of diagnosis	
Initial onset	20
During neoadjuvant therapy	0
During adjuvant therapy	7
During follow-up period	117
Minimally invasive approach	
Yes	99
No	45
R0 resection	
Yes	103
No	41
Disease free interval (median) (months)	0-60 (24)
Frequency of pulmonary metastasectomy	
1	133
2	11
Number of detected pulmonary metastases (median)	1-5 (2)
Summed maximum diameter of detected pulmonary metastases (median) (mm)	3-114 (15)

本组总体3年OS及DFS分别为47.5%（95%CI: 22.0%-75.0%）及38.7 %（95%CI: 15.6%-67.3%）（图 1），其中R0切除组的3年OS及DFS分别为59.2%及41.5%。影响远期生存的单因素分析结果见表 2，OS与DFS共同的影响因素包括肺转移瘤分布（单侧或双侧肺）、切除状态、无病间期、肺转移瘤数目及长径总和。此外，年龄及性别也是OS预后影响因素。多因素分析结果显示，非R0切除[relative risk (RR) 2.78; *P*=0.009]，无病间期 < 1年（RR 2.17; *P*=0.012），肺转移灶数目≥3枚（RR 2.53; *P*=0.019）以及长径总和≥45 mm（RR 2.10; *P*=0.015）是3年OS的独立危险因素；非R0切除（RR 2.14; *P*=0.009），无病间期 < 1年（RR 1.96; *P*=0.013），肺转移灶数目≥3枚（RR 1.59; *P*=0.010）以及长径总和≥45 mm（RR 2.35; *P*=0.008）也是3年DFS的独立危险因素（表 3）。原发灶病理类型、肺转移瘤诊断时机、外科切除方式及肺转移瘤切除术次数则与预后无关。

**1:**
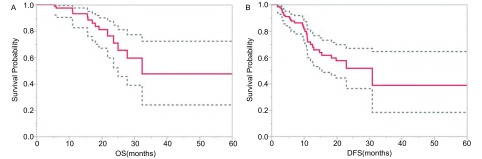
全组144例肺转移瘤切除术患者OS（A）及DFS（B）*Kaplan*-*Meier*曲线 OS (A) and DFS (B) *Kaplan*-*Meier* curves for all 144 patients receiving pulmonary metastasectomy. The red solid line represents the median survival, and the grey dotted line indicates the upper and lower 95%CI

**2:** 单因素预后分析结果 Univariate analysis of prognostic factors

Items	*n* (%)	3-yr			3-yr	
		OS (%)	*P*		DFS (%)	*P*
Age (yr)			0.042			0.573
< 23	78 (54)	49.5			39.7	
≥23	66 (46)	39.1			38.3	
Gender			0.039			0.506
Male	108 (75)	52.5			41.3	
Female	36 (25)	41.5			38.0	
Histology classification			0.085			0.340
Osteosarcoma	107 (74)	46.7			38.1	
Soft tissue sarcoma	37 (26)	42.2			34.9	
Affected laterality			0.036			0.021
Unilateral	97 (67)	54.4			49.1	
Bilateral	47 (33)	42.7			33.9	
Timing of diagnosis			0.188			0.325
Initial onset/During adjuvant Rx	29 (20)	45.7			35.8	
During follow-up period	115 (80)	51.3			38.9	
Minimally invasive approach			0.380			0.510
Yes	99 (69)	52.7			40.2	
No	45 (31)	47.2			37.7	
R0 resection			< 0.000, 1			< 0.000, 1
Yes	128 (89)	59.2			41.5	
No	16 (11)	11.4			0.0	
Disease free interval (yr)			0.030			0.031
< 1	97 (67)	38.3			34.7	
≥1	47 (33)	57.9			40.8	
Frequency of pulmonary metastasectomy			0.083			0.238
1	133（92）	48.9			38.2	
2	11（8）	45.7			34.3	
Number of detected pulmonary metastases			0.009			0.013
1−2	108（75）	59.2			40.4	
≥3	36（25）	24.6			20.9	
Summed maximum diameter of detected pulmonary metastases (mm)			< 0.000, 1			0.002
< 45	117（81）	61.4			39.6	
≥45	27（19）	19.8			11.3	
OS: overall survival; DFS: disease-free survival.						

**3:** 多因素预后分析结果 Multivariate analysis of prognostic factors

Items	RR	95%CI	*P*
3-year OS			
Age, ≥ 23 years	1.12	0.73-6.43	0.294
Gender, female	1.05	0.63-3.43	0.201
Affected laterality, bilateral	1.04	0.71-3.16	0.181
R0 resection, no	2.78	1.56-4.71	0.009
Disease free interval, < 1 year	2.17	1.12-3.69	0.012
Number of detected pulmonary metastases, ≥3	2.53	1.72-2.90	0.019
Summed maximum diameter of detected pulmonary metastases, ≥45 mm	2.10	1.14 -3.75	0.015
3-year DFS			
Affected laterality, bilateral	1.02	0.91 -1.16	0.065
R0 resection, no	2.14	1.10-3.09	0.009
Disease free interval, < 1 year	1.96	1.07-2.77	0.013
Number of detected pulmonary metastases, ≥ 3	1.59	1.14-3.24	0.010
Summed maximum diameter of detected pulmonary metastases, ≥45 mm	2.35	1.12-3.75	0.008
RR: relative risk; CI: confidence interval.			

根据多因素分析结果，26例（18.1%）同时满足四项条件（即R0切除，无病间期≥1年，肺转移瘤数目≤2枚，肺转移瘤长径总和 < 45 mm）的“预后良好”组患者在随访期间（中位随访时间34个月，6个月-60个月）仅1例（3.8%）死亡。与之相比，期间13例（11.0%）“预后不良”组患者死亡。“预后良好”组患者3年OS为80.0%，而“预后不良”组仅为35.8%（*Log*-*rank* *P*=0.071）；两组患者3年DFS分别为85.0%及29.1%（*Log*-*rank* *P*=0.043）（图 2）。

**2:**
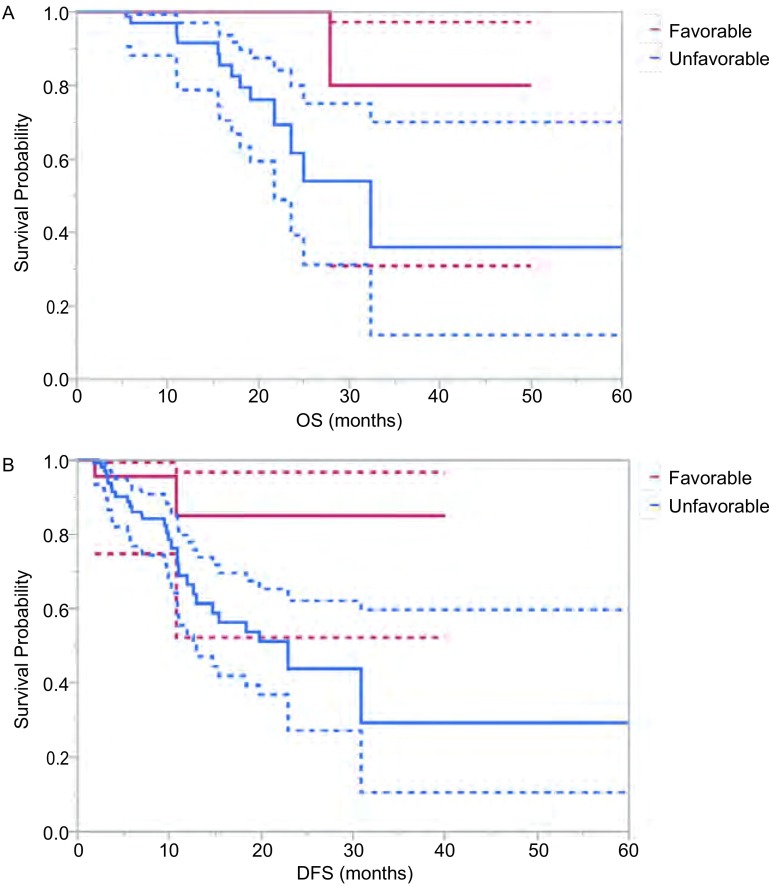
“预后良好”（Favorable）与“预后不良”（Unfavorable）两组患者OS（A）及DFS（B）*Kaplan*-*Meier*曲线 *Kaplan*-*Meier* curves comparing OS (A) and DFS (B) between the favorable (red line) and unfavorable (blue line) groups. The solid line represents the median survival, and the dotted line indicates the upper and lower 95%CI

## 3 讨论

由于骨与软组织肉瘤发病率低，肺转移病例数相对更少难以集中，既往、现在及将来又难以进行所谓随机对照试验，主要证据来源于大样本回顾性研究结果，但是各自存在诸多不一致性。目前仅有的四项大样本研究，分别如下：Briccoli等^[25]^意大利Rizzoli骨科中心323例（1985-2005）骨与软组织肉瘤肺转移的外科治疗结果提示，切除状态及再次复发时间与5年无进展生存率相关。Blackmon等^[26]^美国M.D. Anderson癌症中心234例（1998-2006）肉瘤肺转移患者的预后危险因素包括切除状态、肺转移瘤手术次数及无病间期。Harting等^[27]^美国德克萨斯州医学中心99例（1980-2000）骨肉瘤肺转移的术后结果显示，术前化疗反应、肿瘤细胞坏死比例及无病间期对于预后的影响较肿瘤负荷更加显著；Smith等^[28]^美国Roswell Park癌症中心94例（1976-2000）软组织肉瘤肺转移患者术后生存结果提示切除状态及无病间期是独立的预后影响因素。尽管四项大样本回顾性研究结果均提示切除状态及无病间期是独立的预后影响因素，但是由于研究时间跨度较大，同一组内外科术者及治疗模式之间的差异均存在诸多局限性，使得数据结果难以广泛应用。

同北京积水潭医院骨肿瘤科，集中有世界上骨与软组织肉瘤病例数量最多的专科，组成肺转移性骨与软组织肉瘤诊疗多学科协作组，使得本组肺转移瘤病例数在国内乃至国际均属于大宗报道。通过回顾性分析144例骨与软组织肉瘤肺转移患者的预后因素，发现切除状态，无病间期，肺转移瘤数目及长径总和是3年OS及DFS的独立预后影响因素。根据上述影响因素确定的“预后良好”组患者的预后优于“预后不良”组，但其中仅DFS差异具有统计学意义（*Log*-*rank* *P*=0.043），OS差异虽然未达到统计学意义（*Log*-*rank* *P*=0.071），但是也接近0.05临界值。

本组虽然仍为回顾性研究，但是具有如下特点：①作为国内最大骨与软组织肉瘤诊疗中心，原发灶的治疗策略及方案细节由同一骨肿瘤专业组制定完成，其中骨肉瘤规范治疗模式为术前新辅助化疗+原发灶根治性切除术+术后辅助化疗^[29]^，软组织肉瘤规范治疗模式为根治性外科切除，若术后存在高风险因素（肿瘤直径>5 cm，病理分级较低或合并肺转移）则需联合辅助化疗^[30]^；同时肺转移瘤由同一胸外科专业组完成肺切除术；②本研究时间跨度从2007年至2015年，无论原发病诊疗策略、技术及肺转移瘤切除理念原则均比较统一，这对于数据质量及证据水平均有所提高，其它国际大宗病例报道无法比拟。

肺转移瘤长径总和是较新采用的预后因素，既往曾在结直肠癌肺转移研究中应用^[31]^，但是转移性肉瘤患者中尚未深入研究^[32, 33]^。肺转移瘤长径总和可反应肿瘤负荷。Cadili等^[34]^报道恶性黑色素瘤患者前哨淋巴结总体大小与肿瘤特异性OS显著相关。Iwata等^[35]^证明肺转移瘤总体积是骨肉瘤肺转移术后独立的预后影响因素。Kendal^[36]^通过贝叶斯理论建立的寡转移数学模型预测隐匿性病灶，其中就将肺转移瘤数目、总体大小及无病间期因素纳入模型公式中。本研究中同样发现肺转移瘤长径总和与预后有关，多因素分析确定其与肺转移瘤数目同是独立的预后影响因素，因此肿瘤负荷评估应兼顾数目与体积两个因素。

本研究存在的主要不足为回顾性研究不可避免的偏倚问题；缺少非手术治疗组的预后数据进行比较；纳入分析的软组织肉瘤患者以四肢来源占多数，躯干来源占少数，未包括体腔内来源病例。

综上所述，积极行肺转移瘤外科治疗有助于改善骨与软组织肉瘤肺转移患者的长期预后。R0切除，无病间期时间较长，转移瘤数目较少及长径总和较小是本组患者良好的预后因素。
